# Isolated severe mitral stenosis as an uncommon manifestation of cardiac amyloidosis

**DOI:** 10.1186/s44348-024-00013-6

**Published:** 2024-08-02

**Authors:** Soongu Kwak, Sojung Lim, Yong-Jin Kim, Hyung-Kwan Kim

**Affiliations:** 1 Department of Internal Medicine, Seoul National University Hospital, 101 Daehak-Ro, Jongno-Gu, Seoul, 03080 Korea; 2 Cardiovascular Center, Seoul National University Hospital, Seoul, Korea; 3 Department of Pathology, Seoul National University Hospital, Seoul, Korea

**Fig. 1:**
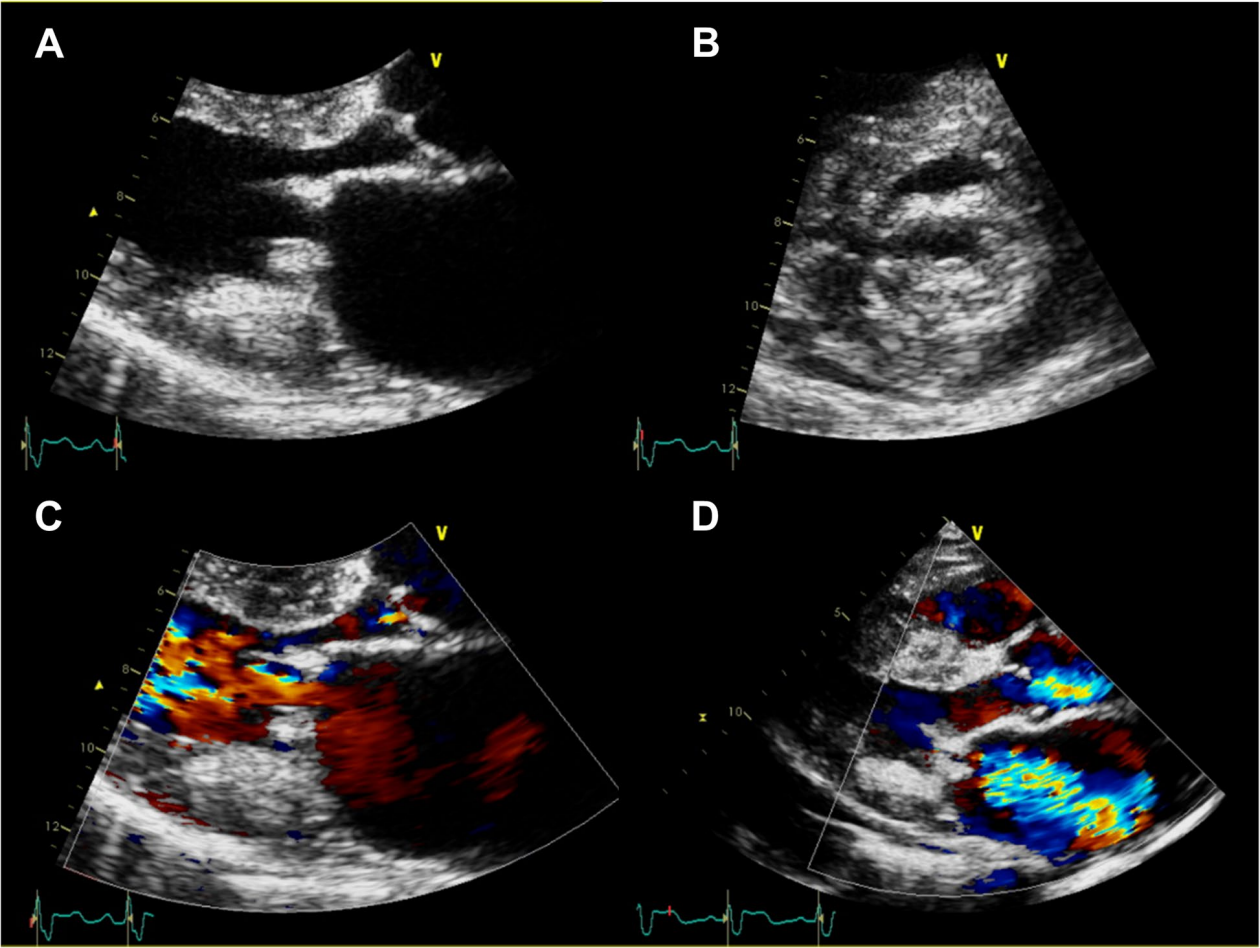
Parasternal view on transthoracic echocardiogram. **A** Zoomed view of parasternal long axis view, demonstrating thickened mitral valve leaflets. **B** Parasternal short axis view demonstrating thickened mitral valve leaflets and restricted valve opening. **C** Accelerated mitral inflow during diastole in the color Doppler, indicating severe mitral stenosis. **D** Concomitant mitral regurgitation observed in the color Doppler

**Fig. 2:**
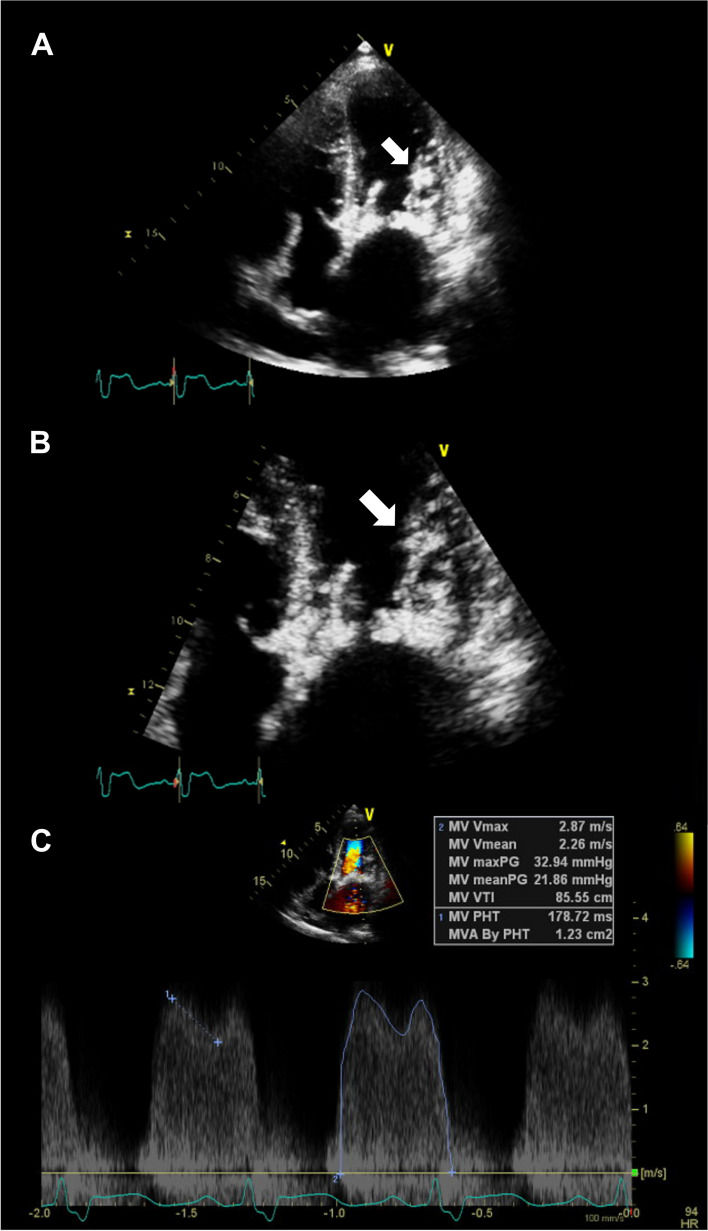
Apical view on transthoracic echocardiogram. **A** Thickened MV with subvalvular mass-like materials (white arrow). **B** Zoomed view of the MV with subvalvular mass-like materials (white arrow). **C** Continuous wave Doppler recordings of mitral inflow with a transvalvular pressure gradient of 21.9 mmHg, suggesting severe mitral stenosis. maxPG: maximum pressure gradient, meanPG: mean pressure gradient, MV: mitral valve, MVA: mitral valve area, PHT: pressure half-time, Vmax: maximum velocity, Vmean: mean velocity, VTI: velocity time integral

A 51-year-old male visited an outpatient clinic with shortness of breath for the last 4 months. He had been diagnosed with stage 4 chronic kidney disease with no verified etiology. Diastolic rumbling murmur was auscultated at the left substernal border and apex. The electrocardiogram showed sinus rhythm with a right bundle branch block, without low QRS voltages. A transthoracic echocardiogram revealed severe mitral stenosis (MS) with concomitant mild-to-moderate mitral regurgitation (Fig. 1, Movie 1), with mitral valve (MV) orifice area measured as 1.27 cm^2^. Both MV leaflets were heavily thickened with severe calcification (Movie 2). Notably, a subvalvular non-mobile mass-like lesion was observed beneath the posterior MV (Fig. 2, Movie 3). No significant abnormalities were observed in other valves. Left ventricular wall thickness was mildly increased to 13 mm, showing concentric hypertrophy. Both serum and urine protein electrophoresis identified M-protein and a bone marrow examination confirmed light-chain amyloidosis. However, the endomyocardial biopsy was negative for cardiac amyloidosis.

**Fig. 3:**
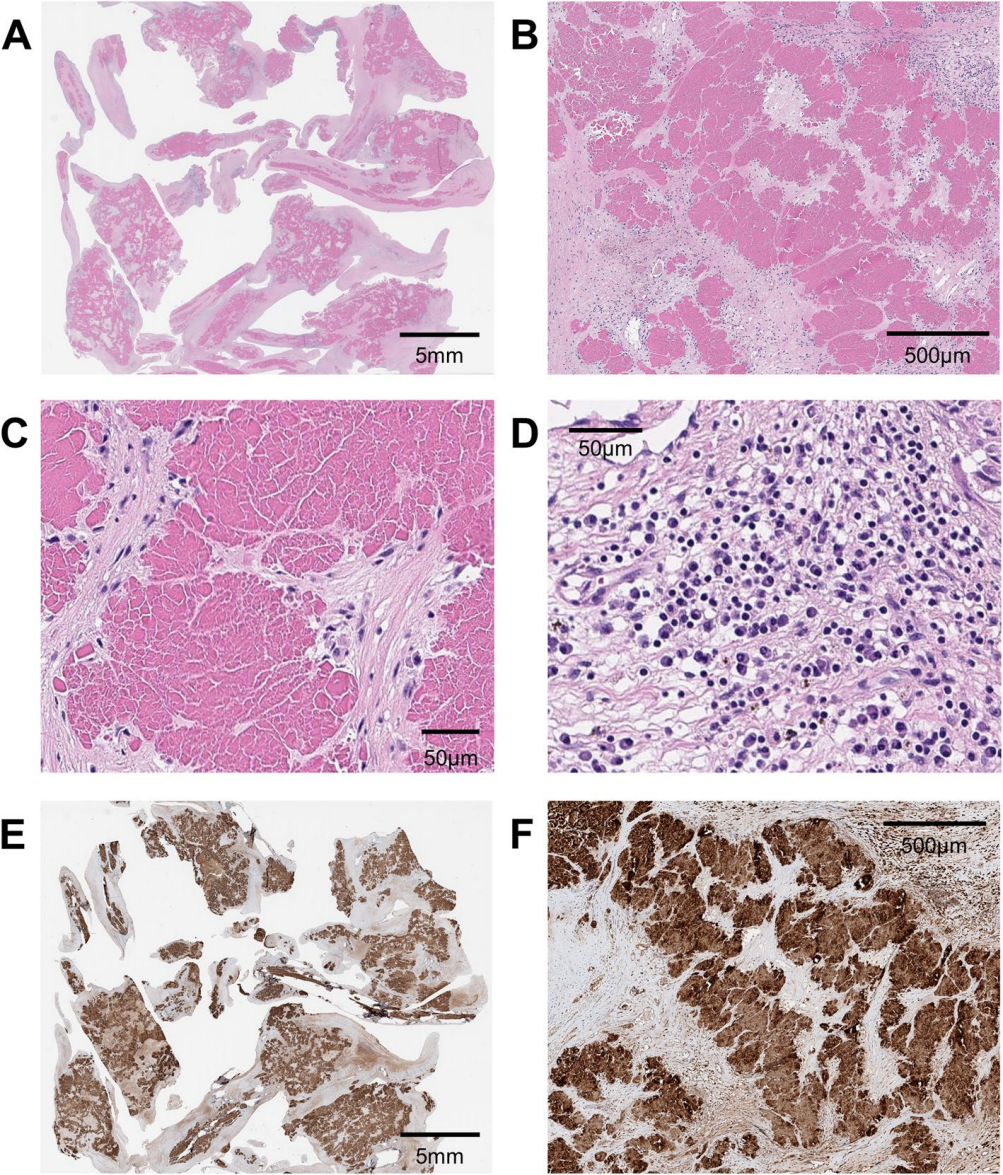
Pathological findings from the surgically excised mitral valve. **A** Low-magnification microscopic view of the excised mitral valve. **B** and **C** Diffuse deposition of abnormal amorphous material on the mitral valve. **D** Lymphoplasmacytic cell infiltration on mitral valve. **E** and **F** Strongly positive lambda light-chain immunohistochemical staining

Surgical MV replacement with a mechanical valve was performed. Pathological findings of the excised valve showed a diffuse deposition of amorphous eosinophilic material on the MV (Fig. 3). Microscopically, abundant lymphoplasmacytic cell infiltration was found with vessel proliferation and inflammation, which was not indicative of rheumatic changes. Strongly positive lambda light chain immunohistochemistry was observed on the MV. However, there was no evidence of amyloidosis in the co-excised myocardium. After MV replacement, he was followed up for 5 years without adverse events, during which autologous stem cell transplantation for amyloidosis and kidney transplantation were performed.

While amyloid deposition can occur on the valves in cardiac amyloidosis, it typically manifests as mild valve thickening, and severe valvular dysfunction is known to be very rare [1–5]. We here present a rare case of severe MS caused by the direct invasion of light-chain cardiac amyloidosis into the MV, as an initial manifestation of cardiac amyloidosis.

### Supplementary Information

**Additional file 1: Movie 1. **Severe mitral stenosis with mild-to-moderate mitral regurgitation was observed in color Doppler mode.

**Additional file 2: Movie 2. **A transthoracic echocardiogram revealed thickened mitral valve leaflets.

**Additional file 3: Movie 3. **A thickened mitral valve with subvalvular mass-like materials was identified in the apical 4-chamber view.
